# Metabolic ecology of microbiomes: Nutrient competition, host benefits, and community engineering

**DOI:** 10.1016/j.chom.2025.05.013

**Published:** 2025-06-11

**Authors:** Erik Bakkeren, Vit Piskovsky, Kevin R. Foster

**Affiliations:** 1 Sir William Dunn School of Pathology, University of Oxford, Oxford OX1 3RE, UK; 2 Department of Mathematics, University of Oxford, Oxford OX2 6GG, UK

## Abstract

Many plants and animals, including humans, host diverse communities of microbes that provide many benefits. A key challenge in understanding microbiomes is that the species composition often differs among individuals, which can thwart generalization. Here, we argue that the key to identifying general principles for microbiome science lies in microbial metabolism. In the human microbiome and in other systems, every microbial species must find ways to harvest nutrients to thrive. The available nutrients in a microbiome interact with microbial metabolism to define which species have the potential to persist in a host. The resulting nutrient competition shapes other mechanisms, including bacterial warfare and cross-feeding, to define microbiome composition and properties. We discuss impacts on ecological stability, colonization resistance, nutrient provision for the host, and evolution. A focus on the metabolic ecology of microbiomes offers a powerful way to understand and engineer microbiomes in health, agriculture, and the environment.

## Introduction

Host-associated microbiomes are important for health, agriculture, and the environment.^1,2^ The exact composition of a given microbiome can also be critical for its effects on a host. In our own gut microbiomes, strain turnover within a single species can be the difference between life and death should a virulent *Escherichia coli* strain replace a non-pathogenic one.^3^ Because of the potential for such effects, there is great interest in the determinants of microbiome composition. The challenge is that microbiomes often contain large numbers of different strains and species. When these species affect each other’s growth and reproduction, they form complex ecological networks where the potential for context dependence can make it hard to identify general patterns and principles.

The field of ecology has provided tools to study how species properties and ecological interactions shape microbiomes.^4^ For example, ecological network modeling demonstrates that microbiomes full of cooperative species, which reciprocally promote each other’s growth, can be much less stable than competitive microbiomes. This effect arises because cooperation can generate destabilizing positive feedbacks.^5^ In line with such general predictions, cooperative interactions within systems like the human microbiome appear to be relatively rare.^6^ However, this type of network modeling is less useful for making specific predictions about a particular microbiome community, such as whether a certain strain will persist, because precise predictions rest upon measuring the entire ecological interaction network. These measurements are challenging and are best done via large experiments that culture microbes, which is not possible for many species that will not grow in the lab. Furthermore, even if experiments are possible, an interaction between two species can change as species are added or lost from a community. This second point is important, as many systems, including the human microbiome, differ in composition among individual hosts, to the extent that each microbiome can be a unique signature of the individual that carries it.^7,8^

**Figure 1:**
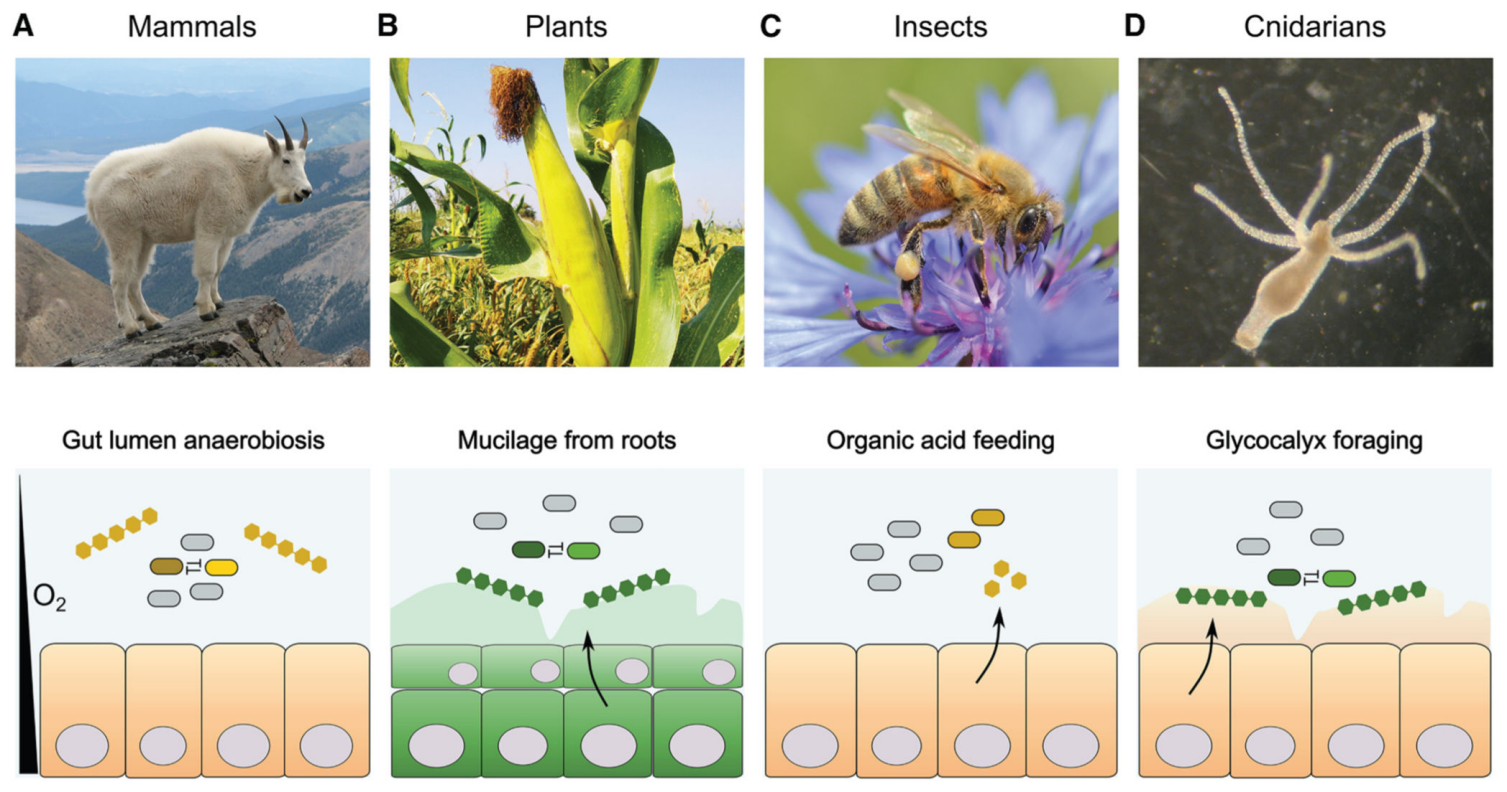
Nutrient competition among microbes is important within diverse microbiomes (A) Many mammals, such as humans, but particularly herbivores, such as cows, mice, and likely the mountain goat (image credit: Darklich14/Wikimedia Commons [CC BY-SA 4.0]), maintain an anaerobic environment in the gut to promote fermentation and the production of short-chain fatty acids like butyrate that feed host colonic cells (beige cells).^11^ Members of the microbiome (yellow rods) compete over available sugar polymers, like fiber (yellow hexagon chains). (B) Plant species such as maize (image credit: Anil/Pixahive [CC0]) produce mucilage from root cap cells (green cells), a gelatinous layer of polysaccharide, proteins, and lipids, which provides carbon sources (green hexagon chains) for competing microbes (green shaded rods).^16,22^ (C) The honeybee (image credit: Clément Bardot/Wikimedia Commons [CC BY-SA 4.0]) creates a nutrient niche for the bacterium, *Snodgrassella alvi* (yellow rods), by the release of organic acids (yellow hexagons) into the gut.^21^ (D) A carbohydrate-rich glycocalyx layer produced by Hydra (image credit: Corvana/Wikimedia Commons [CC BY-SA 3.0]) acts both as a barrier and a nutrient source (green hexagon chains) for competing microbes (green rods).^17^ The glycocalyx layer of Hydra is analogous to mucins of the mammalian gut and is made of glycoproteins and glycolipids, which are secreted by ectodermal epithelial cells.^17^

The core metabolic processes that occur within host-associated microbiomes have the potential to be much better conserved than their species compositions.^9,10^ In humans, the distal gut microbiome is characterized principally by the breakdown of complex carbohydrates and their conversion to short-chain fatty acids, which are taken up by host colonocytes (gut epithelial cells).^11,12^ Moreover, there is growing empirical evidence from diverse host-microbiome systems that the ability to utilize particular nutrients—such as carbon sources,^1,11,13–23^ nitrogen sources,^24–26^ and iron^25,27^—is central to ecological success and ecological interactions within microbiomes (Figure 1). The conservation and importance of metabolism in microbiomes has led to the widespread development and application of whole-genome metabolic modeling methods, and flux balance analysis, which attempt to predict how metabolic reactions within a microbiome change as a function of species composition and the environment.^28^ However, the complexity of these methods makes it much more challenging to study diverse communities of microbes than with general ecological network models.^4^

**Figure 2:**
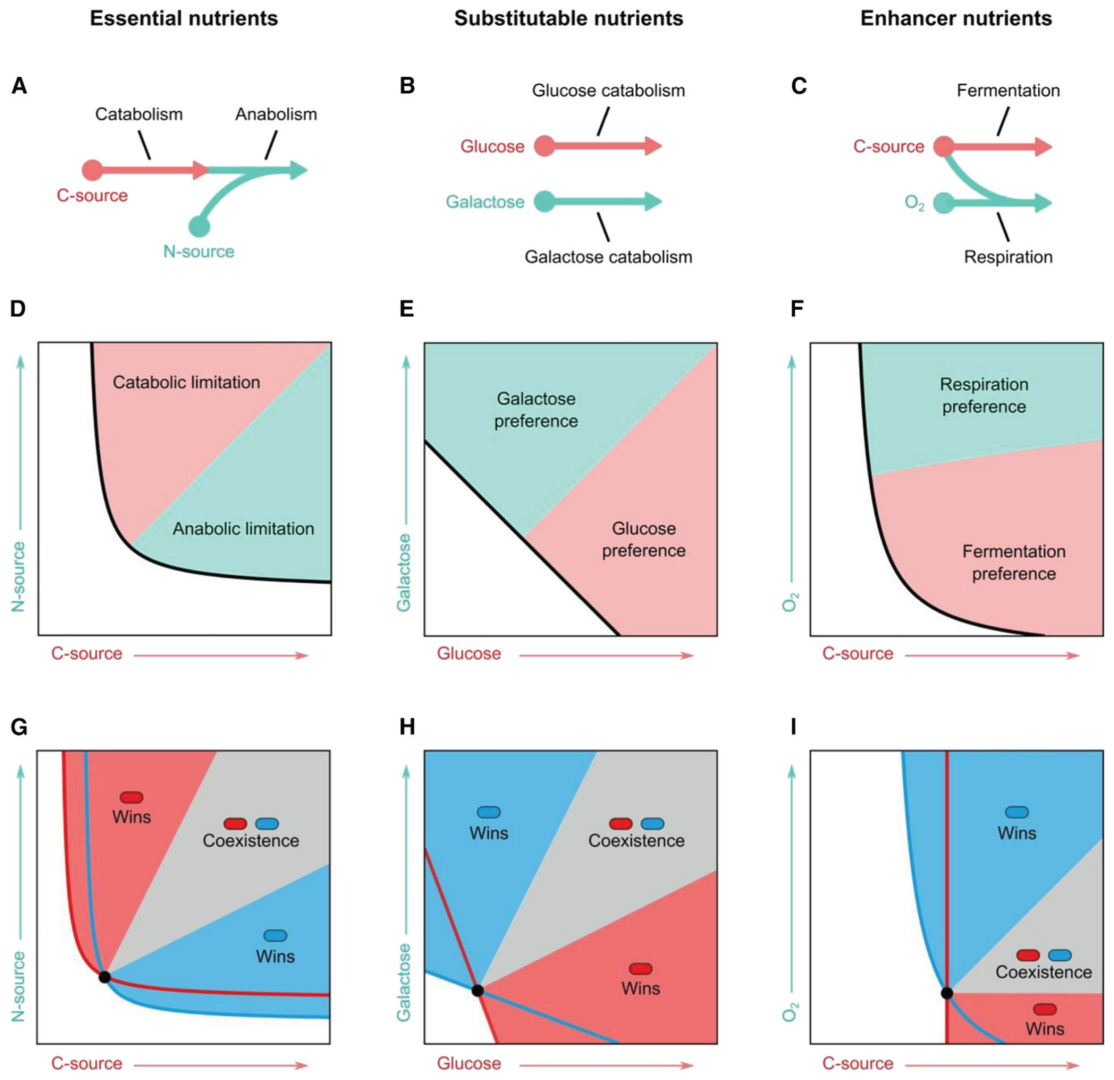
Nutrient classes and their effects on population ecology and species coexistence (A–C) Cartoons to relate the nutrient classes to illustrative metabolic networks: (A) essential nutrients, where a carbon source drives catabolism (pink) and a nitrogen source drives anabolism (green)^31^; (B) substitutable nutrients, where glucose (pink) and galactose (green) can source catabolism along different pathways^32,33^; and (C) enhancer nutrients, where oxygen enhances metabolism by enabling respiration of a carbon source (green) over fermentation (pink). (D–F) Tilman’s graphical approach relates nutrient concentrations to population growth^34^ for (D) essential nutrients, (E) substitutable nutrients, and (F) enhancer nutrients. Here, the black line shows where microbial population growth is exactly balanced by loss (dilution) to give a zero population growth rate. This black line, known as the zero net growth isocline, then separates regions where nutrients cannot support population growth (white region) and regions where growth is sustained by an increased investment into a given metabolic pathway (pink/green regions). The zero net growth isocline does not cross either axis for two essential nutrients, because the population will crash when either is too low (D). By contrast, substitutable nutrients can compensate for one another, which is seen by the intersections of the zero net growth isocline with each nutrient axis (E). When the zero net growth isocline intersects only one nutrient axis, one nutrient is essential while the other can only enhance growth, an enhancer nutrient (F). (G–I) Plots for two species to understand whether coexistence or competitive exclusion is expected with changes in nutrient concentrations for (G) essential nutrients, (H) substitutable nutrients, and (I) in the presence of enhancers. Two microbial species (represented by red and blue rods; their zero net growth isoclines are shown as a solid line of the respective color) can go extinct (white region), competitively exclude each other (blue/red regions), or coexist (gray region), depending on nutrient supply.

Here, we argue that there will be great value in unifying these two complementary sets of approaches, ecological on the one hand and metabolic on the other. We need a metabolic ecology of microbiomes. We take inspiration from the study of animal and plant ecology, where metabolic ecology has long been discussed.^29^ Microbes are particularly well suited to metabolic ecology because of their well-known and general growth laws, such as the Monod equation,^30^ which specifically map metabolism to population growth and ecology (Figure 2). In this view, the set of available nutrients in a microbiome interacts with the metabolism of the microbes to define the composition, properties, and impacts of microbiomes. We focus here on the human microbiome, where there are typically the most data, but the same principles apply to other systems, and we also discuss examples from invertebrate, plant, and environmental microbiomes (Figure 1).

## The availability of limiting nutrients is central to microbiome ecology

The diversity and abundance of nutrients define which microbial species have the potential to grow in a microbiome. However, some nutrients will be much more important than others for a given microbe. Cells require carbon, nitrogen, and energy sources to live in addition to phosphorus, sulfur, oxygen, trace elements like iron, and amino acids or vitamins for auxotrophic species that cannot synthesize their own. These nutrient types are known as essential nutrients because no amount of one can compensate for the absence of another (Figure 2). Even though a bacterium is experiencing a glut of sugars, for example, it can perish if it lacks a nitrogen source to help make proteins.^31^ In particular, it is the growth-limiting nutrient—the nutrient that limits the formation of new biomass—among the set of essential nutrients that is typically the most important for ecology. A large body of empirical work in microbial physiology and genetics has demonstrated the importance of limiting nutrients and metabolism for microbial population biology,^31,32^ including recent studies that mechanistically relate microbes’ metabolic capabilities to their proliferation in gut and plant microbiomes via genome-wide deletion libraries or mutants in metabolic pathways.^14,35–38^

The growth-limiting type of nutrients can vary within and between microbiomes. In mammals, the importance of carbon source limitation is supported by a range of mouse experiments showing that microbial proliferation rests upon supplementation with sugars or sugar alcohols independently of nitrogen,^13,18,19,39^ and there is evidence that carbon sources also can be important in invertebrates,^17,21^ plants,^22^ and soil microbiomes.^15,23^ When metabolized, carbon sources can be used both for energy generation and for anabolism via incorporation of their components into new biomass. However, in the fermentative environment of the anaerobic mammalian gut, it appears that the primary value of carbon sources may come from catabolism and the generation of ATP because, when electron acceptors are present, some species can bloom in response to the increased energy yielded from respiration.^14,20^ Carbon sources are not always growth limiting, however. In the mammalian gut, there is evidence that nitrogen^26^ and iron^27^ can also limit growth. In other environments, nitrogen, iron, and phosphorus limitation can dominate, particularly for photosynthetic species that fix carbon.^24,25^

**Figure 3:**
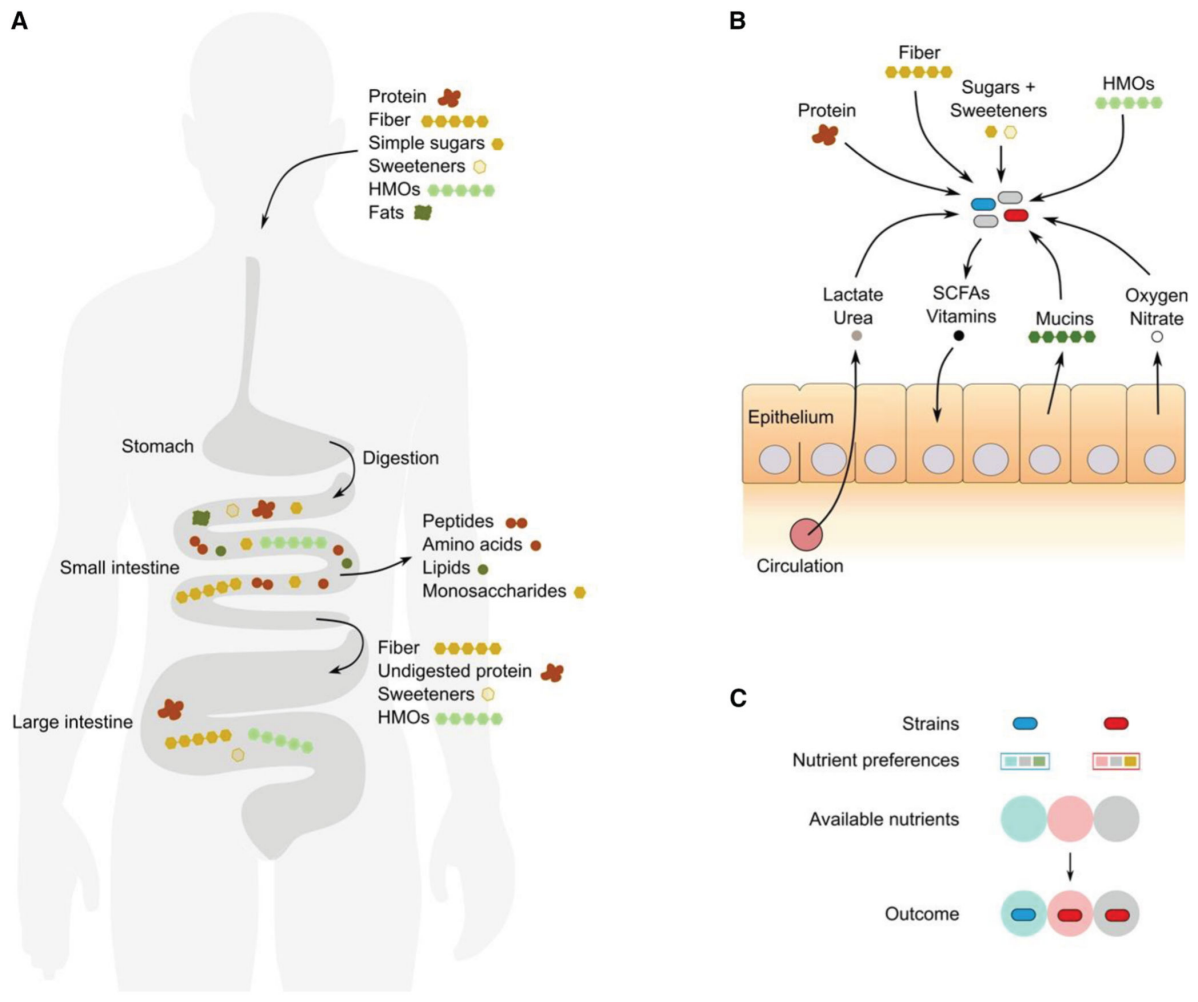
Nutrient niches in the human gut microbiome (A) Ingested food is broken down progressively through the digestive tract, and many nutrients are absorbed by the host before reaching the gut microbiota. Macromolecules are broken down through physical and enzymatic digestion in the stomach and small intestine.^41–48^ HMOs, human milk oligosaccharides. Human silhouette modified from original (credit: Madhero88/Wikimedia Commons [CC BY-SA 3.0]). (B) Microbial niches in the large intestine are defined by the nutrients that remain, as well as nutrients provided by the host. Microbes (red, blue, and gray) compete over these resources to define their niche in the large intestine. SCFA, short-chain fatty acids. (C) Key guiding principle of metabolic ecology. Community composition is shaped by three key factors: (1) the strains present in a community (blue and red rods), (2) their nutrient preferences (colored squares within a rectangle mapping back the preferences to the respective strain), and (3) the available nutrients (e.g., those shown in B; here depicted by shaded circles).

While complexities can arise,^40^ the concept of limiting nutrients offers an important simplification for microbial ecology because it allows one to focus upon one of many types of essential nutrients. However, there can still be a large diversity of potential sources of a limiting nutrient, including many potential carbon sources within the human gut microbiome from diverse complex carbohydrates and other compounds (Figure 3). Such varieties within a class of essential nutrients are known as substitutable nutrients because here an excess of one can compensate for a lack of others (Figure 2). However, while they are in principle interchangeable, not all microbes utilize all substitutable nutrients equally well. The diversity of substitutable nutrients in the limiting class of nutrients, therefore, can define which particular species can proliferate and coexist in a microbiome. Finally, there is also the possibility of what we call enhancer nutrients (also known as “hemi-essential” nutrients^34^), which are not strictly required for growth but can enhance growth on a limiting nutrient (Figure 2).

While the importance of growth-limiting nutrients is clear, which nutrients are actually available is poorly understood for most microbiomes. The human microbiome is one of the best characterized, but even here, there is much we do not know. The nutrient content of many foods is known, but this information alone is not sufficient^41,49^ (Figure 3). Ingested food is rapidly broken down by the host, and many nutrients are absorbed before they reach the microbes.^42,43^ As a result, the diet-derived nutrients in the colon are more likely to be dominated by poorly digestible plant and algal proteins,^44^ complex carbohydrates like fibers,^41,45^ synthetic or natural sweeteners including polyols or sugar alcohols,^46^ and complex milk oligosaccharides in infants.^47^ The simple sugars and amino acids that are commonly used in bacterial growth media, therefore, can be scarce in the colon, where microbial densities are the highest.^43^ This said, monosaccharides have been measured in the cecum of germ-free mice^48^ and are liberated by microbial digestion of more complex molecules, which can fuel the growth of some pathogens.^50^

In many microbiomes, nutrients are also released from the host epithelium.^9^ In mammals, nutrients in the host’s circulation appear to rarely reach the gut lumen, although there is evidence that circulating lactate and urea are used as carbon and nitrogen sources by gut microbes, respectively.^49^ An important source of host-derived nutrients is mucins, which are produced by goblet cells in the intestinal lining. These heavily glycosylated proteins are the principal component of mucus and act as a protective barrier. However, they are also a source of sugars and other compounds, including fucose, sialic acid, galactose, *N*-acetylgalactosamine, and *N*-acetylglucosamine, which are liberated by mucin-degrading microbes and widely used.^51^ Host-provided nutrients can be even more important outside the gut. In the vagina, host-derived glycogen and mucins are the key nutrient sources for the microbiome.^52^ Plants also release large quantities of carbohydrates from the roots,^16^ which include the production of mucilage, a mucin analog, which can feed microbes^22^ (Figure 1).

The available nutrients within a microbiome also can change over time. Soil and rhizosphere microbiomes experience large shifts in nutrient availability and microbial metabolism with the changing weather and seasons. Floods, for example, can drive an increase in microbes engaged in nitrogen metabolism in soil microbiomes due to decreased nitrogen uptake by plants and increasing availability of nitrogen sources for microbes.^53^ In the gut microbiome, antibiotic treatments that reduce microbial numbers can both increase nutrient availability for the microbiota and starve host epithelial cells of microbe-derived nutrients, which promotes inflammation.^54^

## Microbes have evolved diverse metabolisms to harvest nutrients

The available nutrients in a microbiome, therefore, are fundamental for its properties. However, which of the incoming species establish will also depend upon their metabolism and resource requirements (Figures 1 and 3). Here, microbes have evolved a wide range of strategies to harvest and use nutrients. At the extremes, autotrophic species generate complex organic compounds via energy from inorganic sources, including light and inorganic chemical reactions, while heterotrophic species consume organic compounds for both anabolism and energy via production of ATP and reducing equivalents such as NADH.

Among heterotrophic species, a key distinction is whether microbes can respire in addition to performing fermentation. Respiration requires an electron acceptor such as oxygen, which enables a cell to generate far more energy than via fermentation because energy sources can now be oxidized more efficiently and completely. A single glucose molecule, for example, can generate roughly 32 molecules of ATP via aerobic respiration compared with a measly 2 ATPs from fermentation.^55^ For this reason, electron acceptors are powerful enhancer nutrients that can greatly influence growth on essential nutrients without themselves being essential for growth (Figure 2). The electron acceptors that can allow respiration are diverse and include nitrate, iron, and sulfur, but the redox potential of each electron acceptor is different,^56^ with oxygen allowing generation of the most energy. The potential for respiration is limited by a lack of oxygen in the gut of many animals. However, some microbes still manage to respire. One way this is done is via fumarate respiration, where microbes like *S*. Typhimurium or *E. coli* can use microbe-derived hydrogen as an electron donor to reduce fumarate to succinate.^14^ Another is dissimilatory sulfate reduction, where sulfate-reducing bacteria reduce sulfate liberated from mucins by species like *Bacteroides thetaiotamicron* to generate H_2_S (hydrogen sulfide), which is then detoxified by the host to produce thiosulfate, an electron acceptor used by species like *Desulfovibrio spp*.^57^

A wide diversity in available nutrients can lead to a similarly diverse metabolism within a microbiota. Many animals eat plants that contain a diversity of complex carbohydrates that the host cannot digest.^58^ In mammals, gut bacteria carry polysaccharide utilization systems to digest these carbohydrates, including many enzymes to liberate monosaccharides from complex glycans, and importers to bring in the digestion products for fermentation. These systems are especially diverse in genera like *Prevotella* and *Bacteroides*,^59^ where species can carry hundreds of carbohydrate-active enzymes. One study of *Bacteroidales* found that some isolates had more than 50 unique carbohydrate-active enzymes relative to over a hundred other isolates studied.^60^ The importance of nutrient diversity is also seen in honeybees, where a mixture of nutrients derived from pollen supports four closely related *Lactobacillus* species, whereas only one species dominates when bees are fed simple sugar water.^61^ In addition to differences in the ability to consume nutrients, metabolic diversity can arise when microbes differ in their nutrient preferences. For example, glucose and lactose can both be utilized by *E. coli*, but the two sugars are not equivalent for a cell. All else being equal, glucose is preferred and therefore consumed first, while genes for metabolism of lactose are repressed. The result is a classic example of catabolite repression, which results in diauxic growth patterns where microbial growth curves show two distinct phases that correspond to the use of each nutrient. However, diauxie is not always seen, and other substitutable nutrients are consumed simultaneously (co-utilization), such as glucose and pyruvate in *E. coli*.^62^

## Metabolic similarity and nutrient competition shape coexistence

A good fit between a microbe’s metabolism and the available nutrients is important for ecological success. However, a complexity arises because the nutrient environment also depends on the growth responses and abundances of the other microbes in a community. Ecological theory can help to understand such complexity, but the typical approaches used in microbial ecology—notably the Lotka-Volterra equations discussed in the introduction—do not explicitly capture nutrients. Instead, we can turn to pioneering work by the plant ecologist David Tilman,^34^ which shows how the availability of essential, substitutable, and enhancer nutrients can interact with the metabolism and abundances of diverse species to determine ecological outcomes^34^ (Figure 2). A key and intuitive result is that, for species to coexist, they must have metabolism that is best suited to different nutrients. In addition, these nutrients must be supplied at sufficient levels for the microbes to realize their niches (Figures 2G–2I). In this way, species coexistence can occur when each is limited by a different essential nutrient (Figure 2G). When all species are limited by the same essential nutrient, a diversity of substitutable nutrients within that essential nutrient class, such as different sugars, also can allow coexistence so long as species again specialize on different ones (Figure 2H). The presence of an enhancer nutrient can further help with coexistence. For example, relatively low levels of oxygen can support the coexistence of anaerobes that use carbon sources through fermentation and facultative anaerobes that require less carbon source due to the use of oxygen as an electron acceptor (Figure 2I).

While exceptions can arise^63^, these ecological principles formalize the intuition that microbial species need diverse and distinct metabolisms to coexist. A similar argument was made by Rolf Freter in his nutrient-niche theory,^64^ which emphasizes that a microbe will only avoid being driven extinct (competitive exclusion) if it can access a limiting nutrient better than any other species in a community (Figure 3C). The importance of metabolic diversity for microbial coexistence is well supported by data. Indeed, the phenomenon of competitive exclusion is also known as Gause’s principle^65^ due to Gause’s famous *Paramecium* experiments that led him to conclude in 1934 that species that share the same resource requirements do not coexist.^66^ In the human gut, bacterial species vary greatly in their potential for strain coexistence, i.e., whether multiple strains of one species occur together in a person.^8^ Consistent with the importance of metabolic diversity, strain coexistence is more likely in species with an enrichment of metabolic genes in their accessory genome (the set of genes in the species that are only carried by some strains). *E. coli* has an unusually large accessory genome, and strains commonly differ in the carbohydrates that they can import and utilize.^67^ As a result, the strength of competition between *E. coli* strains can vary with both strain identity and nutrient conditions. For example, one study found that a combination of two *E. coli* strains will prevent a third pathogenic *E. coli* from colonizing antibiotic-pretreated mice, but neither *E. coli* alone could prevent the pathogenic *E. coli* from colonizing.^68^ This result was explained by the two non-pathogenic *E. coli* using complementary but divergent sets of sugars during colonization, such that only in combination could they starve the pathogenic *E. coli*.^68^

As compared with strains of one species, different species typically overlap less in their metabolism and potential for nutrient competition. Nevertheless, under some conditions, strong competition can occur. Many *Bacteroides* species can degrade both fiber and mucins, while others, like *Akkermansia muciniphila*, are more specialized, in this case on the degradation of mucin O-glycans. When mice are given a fiber-rich diet, *Bacteroides* species will degrade fiber, while *A. muciniphila* forage mucus. However, with a diet that is low in fiber, *Bacteroides species* switch to utilizing mucin and compete with *A. muciniphila*, which erodes the mucus layer.^41^ Moreover, even if nutrient competition between any two species is weak, collective competition effects can be strong.^13^ For example, in soil microcosms, phylogenetically diverse species can protect against an invading strain of *E. coli*, but only when a sufficiently diverse community is present.^15^

## From metabolism to ecological interactions

Ecological interactions among species shape the composition and properties of microbial communities. It is important, therefore, to understand how microbial metabolism shapes the full diversity of ecological interactions within microbiomes.^69^ This requires one to appreciate the impacts of metabolism not only on nutrient competition but also on other mechanisms of ecological interaction, including cross-feeding and interference competition via microbial (particularly bacterial) warfare.

### Niche overlap, nutrient competition, and cross-feeding

As we have discussed, a key principle of metabolic ecology is that the extent of nutrient-niche overlap among strains is a major determinant of ecological interactions and, ultimately, patterns of coexistence. Two strains that overlap perfectly in their nutrient preferences will strongly and negatively affect each other’s growth in a competitive (−/−) interaction. However, metabolic differences between strains can relax this competition, leading to the potential for coexistence and a diversity of potential interaction outcomes ranging from weaker competition (−/−) through amensalism (−/0) to a neutral interaction (0/0) where the strains no longer affect each other’s population dynamics. If metabolism and nutrient competition are central to microbial ecology, therefore, coexisting species are expected to display many weakly negative ecological interactions. This prediction is well supported by a wide range of studies that have cultured bacterial species alone and together to assess their ecological interactions from a range of environments, including soil, invertebrates, and the mammalian gut microbiome.^6,70–72^

However, positive interactions also occur. Here, metabolism is again central because of the widespread potential for cross-feeding, whereby a product made by one species is used by another as a nutrient.^73,74^ Cross-feeding can arise in diverse ways, including many examples where species use the waste products of others, but also cases where the extracellular digestion of a complex molecule by one strain releases monomers that others harvest. Another mechanism is piracy by one strain of another strain’s siderophores that function to chelate metal ions for import.^75,76^ Such inadvertent sharing of nutrients also occurs via host^77^ or microbial cell lysis,^78^ whether driven by phage or other processes, which releases a wide diversity of nutrients into the environment for other strains to use.

Cross-fed nutrients are then diverse^73^ and include fermentation products; degradation products from complex molecules, including mucins, fibers, proteins, and lipids; and also the release of small molecules such as amino acids, vitamins, or essential co-factors that other species cannot produce themselves. In the anaerobic gut, polysaccharide foraging leads to complex cross-feeding networks^59^ where primary degraders liberate oligo- or monosaccharides, which they and primary fermenters convert to partially oxidized metabolic byproducts like fumarate, lactate, or formate. Secondary fermenters then ferment these byproducts to short-chain fatty acids like acetate, propionate, and butyrate, which are taken up by the host.^73^ In environments with high ammonia, such as wastewater, ammonia-oxidizing bacteria oxidize ammonia to nitrite that nitrite-oxidizing bacteria convert to nitrate, which is used as an electron acceptor by yet other microbes.^79^ In these networks, cross-feeding interactions can be strongly positive from producer to consumer. In some cases, a producer may also benefit in return,^80^ but this does not appear to be typical. Instead, both theory and experiments with diverse microbial species suggest that commensal (+/0) or even exploitative interactions (+/−) are more common, where the latter can occur when consumer and producer strains compete over a limiting nutrient.^6,81^

### Microbial warfare

In addition to mechanisms of nutrient competition, microbes have evolved an astonishing diversity of mechanisms to directly harm, kill, or restrict the growth of competing microbes.^82^ Such direct inhibition is known in ecology as interference competition and is the second key form of ecological competition, after resource competition. In bacteria, these competition systems, or “weapons,” include diffusible factors such as protein or small-molecule toxins^83^ and contact-dependent weapons like type 6 secretion systems (T6SS), which function like tiny spearguns that fire into neighboring cells to deliver toxic effector proteins.^84^ The expression of bacterial weapons is typically tightly regulated, which is thought to ensure that they are expressed when most useful. Weapon genes can be upregulated not only in response to cell damage and quorum sensing but also nutrient limitation, which can be a proxy of the intensity of inter-strain competition.^85,86^ Weapon use is expected to be the most beneficial against close competitors, rather than species that use different resources.^85,87,88^ As a result, the level of metabolic similarity between strains can be indicative of the likelihood of them fighting. Consistent with this, many antibacterial toxins, like the colicin toxins of *E. coli*, function by binding to specific receptors on target cells, which restricts their functioning to relatively closely related strains and species that carry the receptors.^89^ However, fighting among closely related strains can also drive the evolution of resistance mechanisms, which renders weapons ineffective.^82,90,91^

Some weapons inhibit a wide diversity of species, including small-molecule antibiotics and the T6SS. Contact-dependent weapons like the T6SS, in particular, function well when a strain is invading its preferred niche and surrounded by competitors.^92^ In practice, then, the proximity of metabolically similar genotypes may mean that these weapons also typically target close competitors. However, evolutionary theory predicts that some microbes can benefit from the aggressive targeting of a wide range of species.^88^ The argument goes: if a strain is abundant enough, it will have the numbers to use a broad-spectrum antimicrobial to globally suppress all surrounding species and reinforce its ecological dominance. In support of this hypothesis, broad-spectrum (but not narrow-spectrum) antimicrobials are commonly upregulated by quorum sensing, i.e., bacteria preferentially use broad-spectrum toxins when they are abundant.^88^ In such cases, the outcome of nutrient competition can be reinforced by a microbial weapon that finishes off a struggling competitor.

While mechanisms of interference and nutrient competition are largely distinct, the ways that microbes use nutrients can strongly influence the regulation and impacts of bacterial weapons.

## Metabolism, host control, and community properties

**Figure 4:**
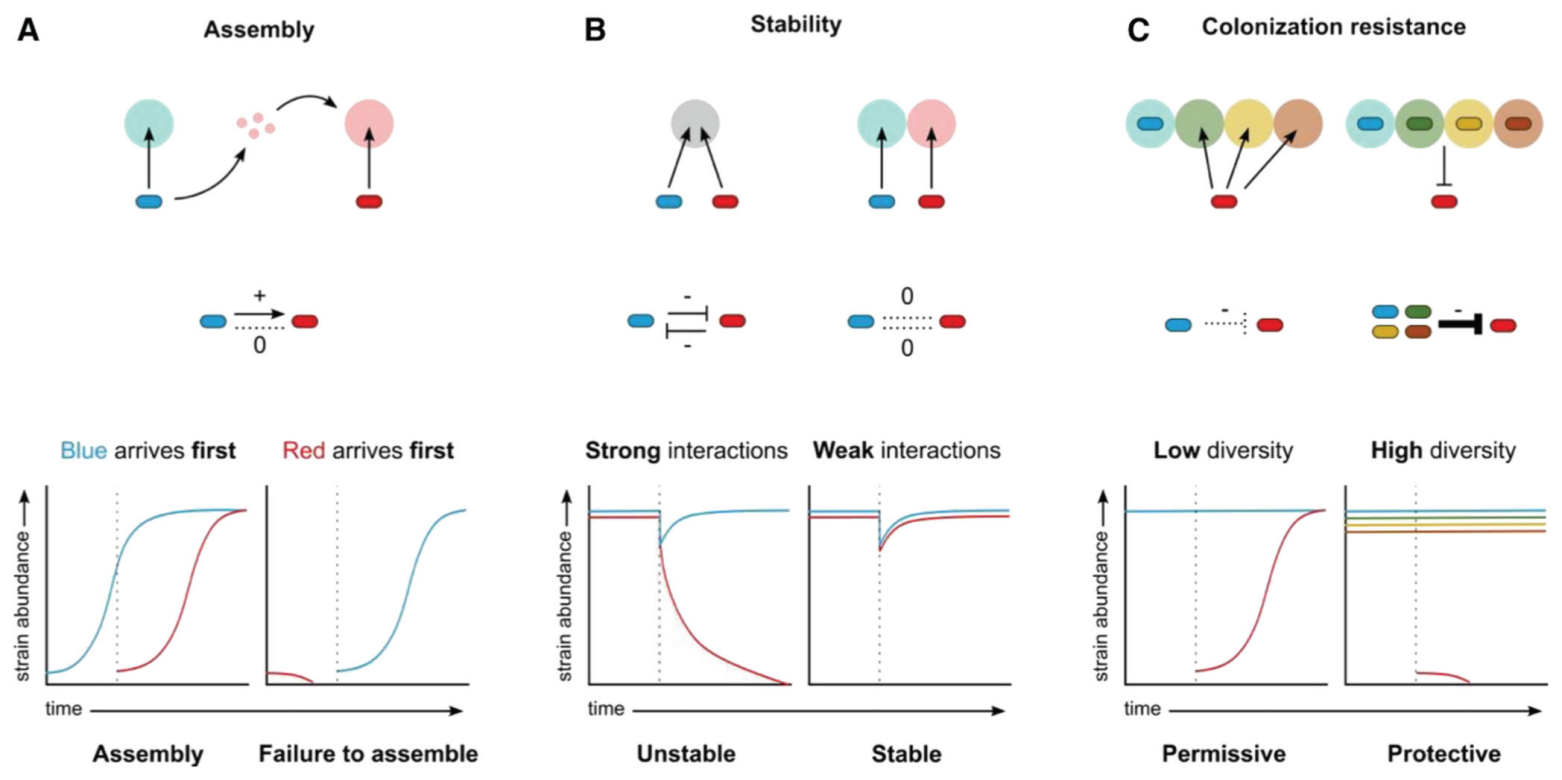
Nutrient utilization shapes ecological interactions and population dynamics The relationship between available nutrients and which microbes can use those nutrients (top row) helps define if microbes interact positively (+), negatively (−), or neutrally (0) (middle row). These interactions determine system-level properties such as how communities assemble^93^ (A), their stability^5^ (B), and their ability to protect against pathogens^13^ (colonization resistance; C), shown by population dynamics plots (bottom row). (A) An example of two species where the blue species utilizes a blue nutrient and produces a red nutrient that the red species utilizes (+/0 interaction). The vertical dotted line indicates the arrival of the second species. If blue arrives first, the community can assemble properly. If red arrives first, then there is no available red nutrient, and assembly fails. (B) An example of two species that compete for the same nutrient (left) or each have their own nutrient (right). This corresponds to a reciprocal negative interaction (−/ −; left) and a neutral interaction (0/0; right). If a community made up of these two interacting species is perturbed (vertical dotted line), the strongly interacting pair may not return to their original state, as one can eliminate the other (left). The neutrally interacting community can re-establish itself without the species affecting each other (right). (C) A low-diversity community (left; blue) can occupy fewer nutrient niches than a high-diversity community (right; blue, green, beige, and orange). This corresponds to a weak negative interaction with an invading pathogen (red) by the low-diversity community (left), but a strong negative interaction against the pathogen provided by the community collectively (i.e., nutrient blocking^13^; right). The pathogen can then only establish itself in a low-diversity community. The time of invasion is indicated by the vertical dotted line.

**Figure 5:**
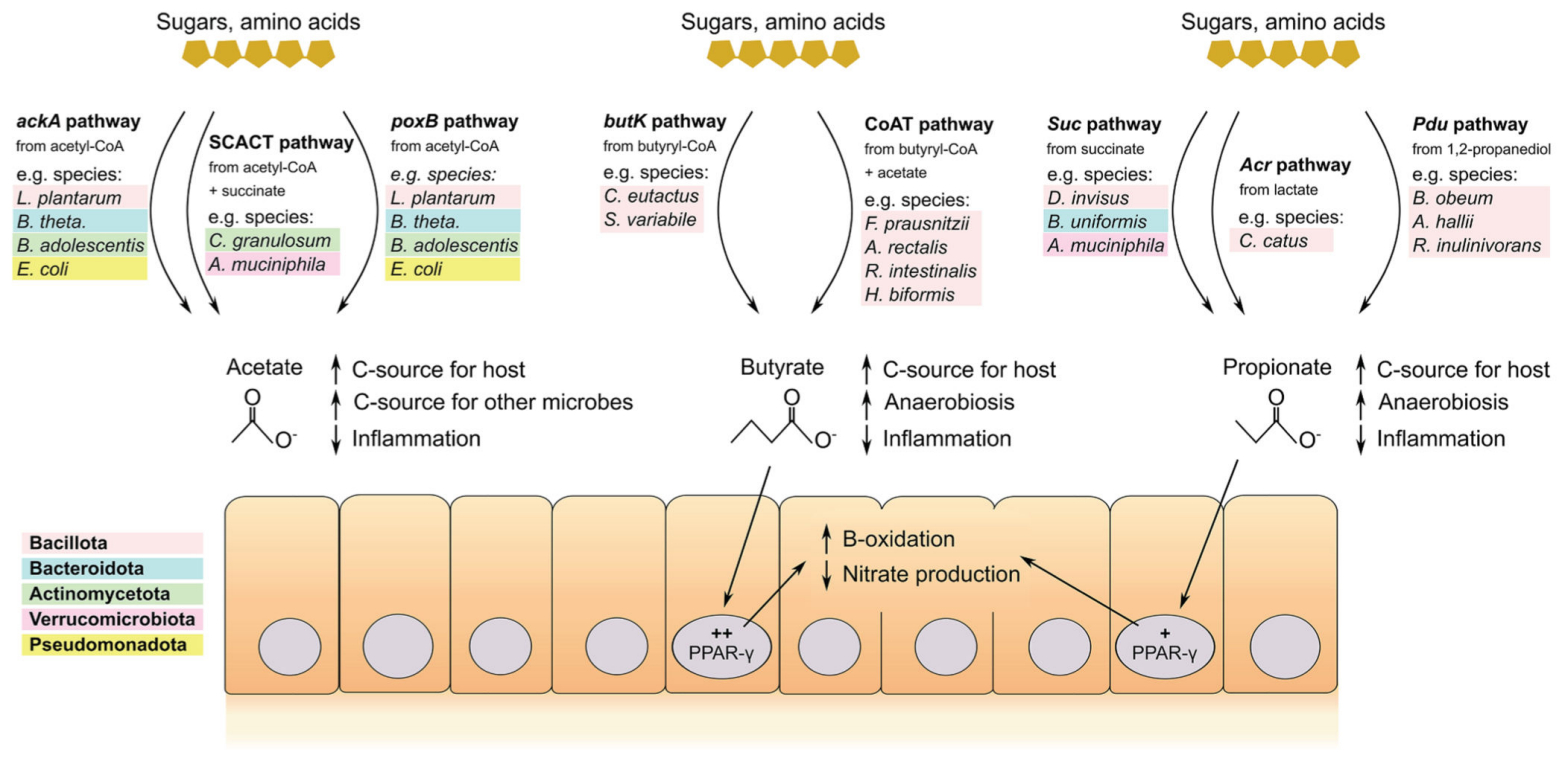
Host physiology selects for conserved metabolism despite wide variation in microbiota composition and nutrients In the mammalian gut, diverse nutrient sources such as sugars (yellow hexagon chains) or amino acids can be used as both carbon and energy sources by diverse microbes. Despite this variability, common outputs from these pathways are acetate, butyrate, and propionate. Acetate is produced from acetyl-coenzyme A (CoA) via 3 main pathways: the acetate kinase (*ackA*) and phosphotransacetylase reactions pathway, the succinyl-CoA:Ac-CoA transferase (SCACT) and succinyl-CoA synthetase pathway, or the pyruvate:menaquinone oxidoreductase (*poxB*) pathway.^98^ Diverse species can perform these reactions, with examples listed and color-coded by phyla^99–105^ (Bacillota, formerly Firmicutes, in red; Bacteroidota, formerly Bacteroidetes, in blue; Actinomycetota, formerly Actinobacteria, in green; Verrucomicrobiota in purple; Pseudomonadota, formerly proteobacteria, in yellow). Acetate serves as a carbon and energy source for colonocytes (beige cells), regulates inflammation, and acts as a carbon and energy source for other microbes via cross-feeding.^98,106^ Butyrate is produced by two main pathways from butyryl-CoA, exclusively by Bacillota: the butyrate kinase (*butK*) pathway and the butyryl-CoA:acetate CoA-transferase (CoAT) pathway.^107^ Propionate is produced by phylogenetically diverse bacteria via 3 main pathways: from succinate via the succinate (*Suc*) pathway, from lactate via the acrylate (*Acr*) pathway, or from 1,2-propendiol via the propendiol (Pdu) pathway.^107,108^ Both butyrate and propionate serve as host energy sources via beta-oxidation, although butyrate provides more energy.^11,12,109^ Butyrate^11^ and, to a lesser extent, propionate^109^ also signal via the transcription factor PPAR-γ to reduce nitric oxide synthase expression and consequently nitrate availability in the gut, as well as shift colonocyte metabolism to beta-oxidation from anaerobic glycolysis. Beta-oxidation consumes oxygen and thus reduces the availability of electron acceptors so that facultative anaerobes do not bloom. Full species names: *Lactiplantibacillus plantarum, Bacteroides thetaiotaomicron, Bifidobacterium adolescentis, Escherichia coli, Cutibacterium granulosum, Akkermansia muciniphila, Coprococcus eutactus, Subdoligranulum variabile, Faecalibacterium prausnitzii, Agathobacter rectalis* (formerly *Eubacterium rectale*), *Roseburia intestinalis, Holdemanella biformis, Dialister invisus, Bacteroides uniformis, Coprococcus catus, Blautia obeum, Anaerobutyricum hallii* (formerly *Eubacterium hallii*), and *Roseburia inulinivorans*.

Microbial metabolism shapes the ecological interactions within microbiomes via impacts on nutrient competition, cross-feeding, and microbial warfare. These interactions, in turn, are central to the properties of communities and their impacts on a host (Figure 4), including (1) assembly and species coexistence,^93,94^ (2) ecological stability,^5,95,96^ (3) colonization resistance against pathogens,^13^ and (4) the production of key metabolites like butyrate that feed the host.^11^ As a result, hosts are expected to benefit from acting as ecosystem engineers that shape both microbiome composition and ways that species interact.^9,97^ Consistent with this expectation, hosts exert control over their microbiomes via diverse mechanisms, including immunity, barrier function, transit, and host feeding behavior (reviewed in Wilde et al.^9^). Central to these processes is control over the nutrients that are available to the microbes,^97^ which can select strongly for particular forms of microbiome metabolism despite variability in species composition^7,8^ (Figure 5).

Host-associated communities often progressively assemble over time as the host develops (Figure 4A). In the gut microbiome, infants are colonized by microbial species at birth, followed by a succession of species as the infant develops physiologically and changes diet from milk to solid food.^110^ *Bifidobacteria* species are dominant in the newborn infant gut, which produces fermentation products that feed butyrate-producing species like *Roseburia spp*., *Faecalibacterium spp*., *Agathobacter rectalis*, or *Anaerostipes spp*.^110,111^ In this way, cross-feeding can generate novel nutrient niches within a microbiome that shapes community assembly and helps render it predictable.^93,112,113^ Moreover, hosts have evolved to influence the process. Mother’s milk provides a diverse range of complex oligosaccharides that *Bifidobacteria* can utilize but, importantly, the baby cannot, which helps to anchor and regiment the assembly process.^93,112^ In other systems, honeybees create a nutrient niche for the bacterium *Snodgrassella alvi* by releasing organic acids into the gut,^21^ and the carbon sources released by plant roots shape their microbiomes^22^ (Figure 1).

Another key property of many microbiomes is the ability of species to bounce back robustly in the face of perturbations, which is known as ecological stability^95,96^ (Figure 4B). Here again, the strength and signs of the ecological interactions among species can be critical for how a community behaves as a system.^95,96^ In addition to limiting cooperation (+/+) with its potential for positive feedbacks, stability can be increased when species interact relatively weakly.^5^ Hosts, therefore, may benefit from segregating or isolating species to limit the strength of nutrient competition and the probability that one species drives another extinct during perturbations.^9^ Structural features like blind-ended crypts in the mammalian gut, for example, can help to isolate strains,^114^ and some hosts house their symbionts in dedicated structures, as occurs in the light organ of the bobtail squid^115^ and the root nodules of legumous plants.^116^ Another important potential mechanism is host provision of nutrients, such as the mucins that feed many of the fermentative bacteria in the mammalian gut.^5^ Nutrient provision has the potential to both create new niches within the microbiota and reduce the strength of competition to render the community more stable. Consistent with such effects, mice that cannot produce the fucosylated glycans that feed many species have a lower diversity gut microbiome than those that can.^117^ Diets with abundant and variable complex molecules that feed the microbiota may also help create a diverse and stable microbiome because species numbers are higher in humans eating fiber-rich hunter-gatherer diets compared to Western diets with highly processed foods.^118,119^

A key benefit of many microbiomes is to protect the host against incoming microbial pathogens. Competition within the human microbiota is central to this colonization resistance, which limits the nutrients available to incoming pathogens, including carbon sources,^13,18,50,120^ amino acids,^121,122^ and iron.^27,123^ Known as “nutrient blocking,”^13^ this effect helps to explain why diverse microbiomes are beneficial for a host, because diversity within a microbiome increases the probability that pathogen nutrient sources are consumed^13^ (Figure 4C). Nutrient competition also promotes colonization resistance in plant microbiomes^124^ and similar effects are seen in environmental microbiomes.^15^ Hosts can benefit from promoting colonization resistance. One striking response is seen after bacterial infections, where mice increase the bile acid taurine in the gut. This response promotes taurine metabolism in the microbes, which releases sulfide and inhibits respiration in key bacterial pathogens.^125^ Other responses are seen while an infection is in process, such as the release of lipocalin-2 to limit the iron available to pathogens.^126^

Many microbiomes also provide nutrients for a host. Herbivores rely on microbial fermentation of plant material (Figure 1), and humans benefit from this too, albeit to a lesser extent^11,12^ (Figure 5). Central to this fermentation is the maintenance of an anaerobic gut by a host.^9–11^ This ensures that, rather than converting ingested food to water and CO_2_ via aerobic respiration, the microbes generate fermentation products, such as butyrate, which can be absorbed by the host.^10,11^ In mice, microbial production of butyrate has been shown to reinforce gut hypoxia by signaling to intestinal cells via peroxisome proliferator-activated receptor-γ (PPAR-γ) to activate beta-oxidation, which consumes large amounts of oxygen and prevents it from leaking into the gut.^54^ By contrast, the absence of butyrate results in fewer regulatory T cells,^127^ which can lead to inflammation and the potential to restructure the microbiome and restore beneficial members.^97^ Similar control over microbiome metabolism is seen in other species, including the maintenance of an anaerobic gut in cockroaches^128^ and the low oxygen levels in the root nodules of legumes that allow rhizobia to both fix nitrogen and respire.^129^ However, some pathogens have evolved strategies to circumvent such control and reshape their microbiome’s nutrient environment for their own benefit.^130^ A key example from the anaerobic mammalian gut is *Salmonella enterica* Typhimurium (*S*. Typhimurium), which actively promotes inflammation^131,132^ and thereby generates electron acceptors, including oxygen, nitrate, and tetrathionate, that allow it to respire and bloom. More generally, there are many examples where pathogens manipulate host physiology, and even development, to promote nutrient access, including diverse microbes that promote gall formation in plants.^133^

## Evolutionary consequences of metabolic ecology

The available nutrients within a microbiome strongly shape the population biology, species interactions, and community ecology of the resident microbes. Metabolic ecology is also important for evolution within microbiomes via effects on genetic variability, natural selection, and the evolution of adaptations that shape metabolism and species interactions.

### Population genetics within microbiomes

**Figure 6:**
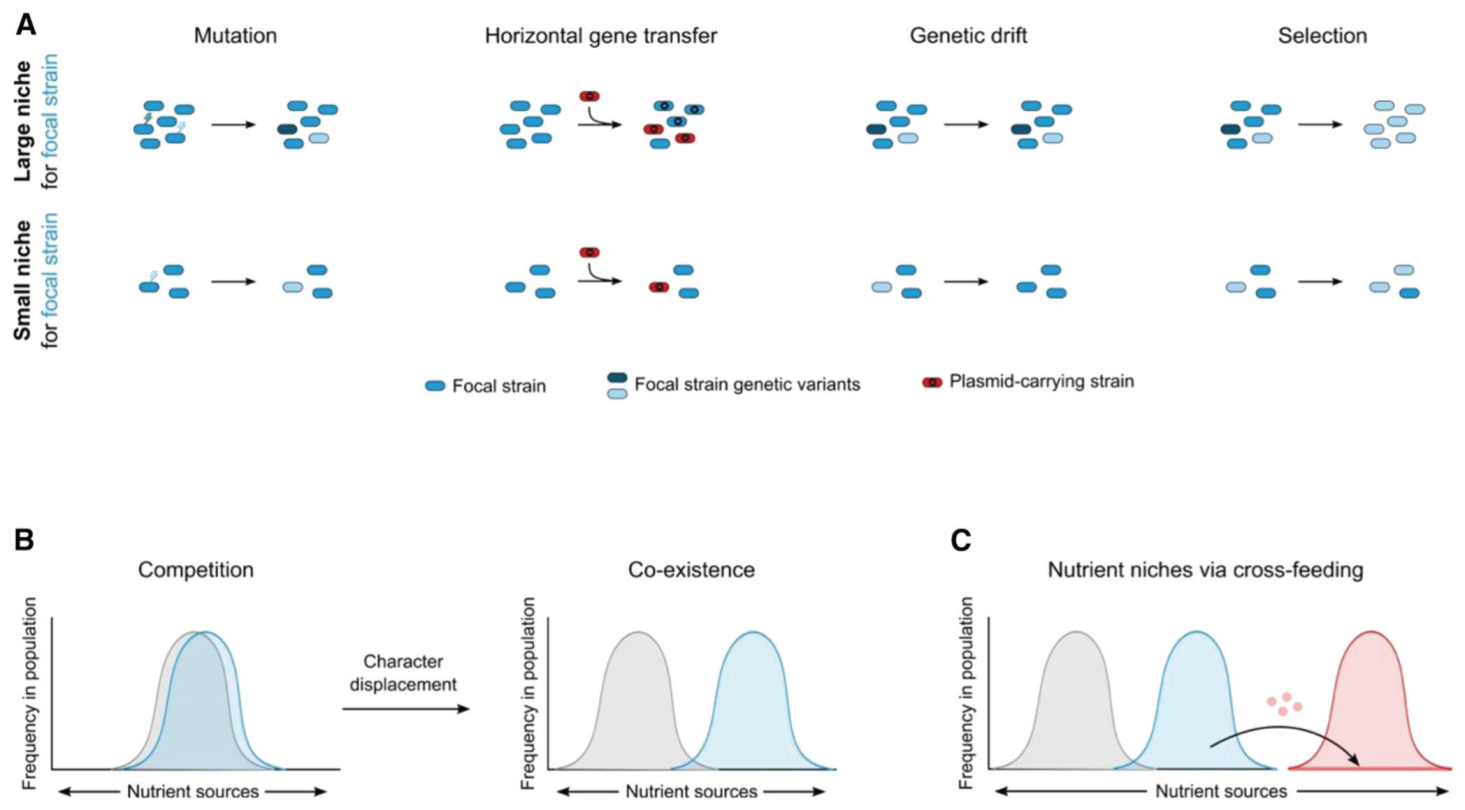
Evolutionary consequences of metabolic ecology (A) Population genetics: nutrient supply and ecological competition with other species can define the available nutrient niche for a species and thereby its population size. Large population size can increase genetic variation because of increased probabilities of mutation and horizontal gene transfer. Large population sizes also reduce the probability that genetic variation is stochastically lost via genetic drift, which is another reason that small population sizes can display less standing variation. However, another consequence of reduced stochasticity in large population sizes is that natural selection is more powerful. As a result, a beneficial allele is more likely to sweep to fixation along with genetically linked loci, which is a process that can reduce genetic variability and act against the other processes discussed. (B) Character displacement. A key expected consequence of natural selection on strains under nutrient competition is for one or more to diverge in their metabolism and nutrient use, with the consequence that competition is relaxed and coexistence is more likely. (C) Niche creation via cross-feeding. Natural selection to compete in the microbiome can result in metabolism that generates byproducts, which may then be used by other species as nutrients (red circles creating a niche for a red species). In this manner, one-way positive interactions and cross-feeding can readily evolve.

Nutrient availability will shape the genetic variation within species that is required for natural selection to occur. When nutrients become scarce, population size can plummet, and with it, the level of standing genetic variation. This drop occurs because the probability of new mutations is now reduced but also because stochasticity at low population sizes can cause existing genetic variants to be lost (genetic drift)^134^ (Figure 6A). The large population sizes of many microbes might suggest that these effects are rare. However, in hosts with diverse microbiomes, the population sizes of individual species can be relatively low due to ecological competition, particularly in hosts with a small body size.^135^ Moreover, the standing genetic variation in a population is primarily determined by the lower bounds of its size.^136^ Fluctuations in nutrient supply via diet and other effects, therefore, have the potential to greatly reduce the variation available to natural selection within a species. Indeed, a study following a natural dominant clone of *E. coli* in the human gut found evidence of genetic drift.^137^ Population bottlenecks can also occur when a strain first arrives and faces competition with resident species (a founder effect).^138^ Moreover, when microbes grow in structured communities like biofilms, genetic diversity can be massively reduced due to local competition and stochasticity at the growing edge.^139,140^ As a result, strong genetic drift can occur in communities of many billions of cells, an effect further amplified with nutrient scarcity.^141^ Such structured growth occurs in some microbiomes, including those on teeth and the tongue.^142,143^ However, the importance of structured growth remains unclear in the mammalian gut microbiome, where peristalsis can mix up microbial genotypes.^144^

Genetic variation in microbes is also influenced by horizontal gene transfer, which can occur via plasmids, bacteriophages, and the uptake of environmental DNA. Horizontal transfer enables a strain to rapidly acquire new functions and is central to antibiotic resistance and virulence^145,146^ but can also involve metabolic genes that influence nutrient use.^147^ The probability of transfer events can be strongly influenced by nutrient availability and population size. Here, there needs to be sufficiently high population sizes in two strains, both donor and recipient,^148^ which can occur when both use abundant nutrients (Figure 6A) or during a bloom associated with other factors like inflammation in the gut.^149^ A constraint is that, while horizontal transfer is most likely among closely related strains,^150^ this is also where competitive exclusion is most likely. As a result, a difference in nutrient preferences between otherwise similar strains is expected to maximize the probability of transfer events.^39^

When a new beneficial allele or gene occurs via mutation or horizontal transfer, natural selection can increase its frequency. However, the strength of natural selection is again linked to nutrient availability because genetic drift can overpower natural selection at low population sizes and limit adaptive evolution (Figure 6A). The power of natural selection on species in microbiomes is not yet clear,^138^ but there is evidence of positive selection in mouse experiments.^151–153^ In addition, rapid selective sweeps have been documented in some human gut species, including evolution in polysaccharide utilization loci that suggests adaptation to prevailing nutrient conditions.^154^ Genomic signatures also suggest the widespread occurrence of purifying selection in the human microbiome, which helps to eliminate deleterious alleles.^135,155^ However, as discussed, genetic drift and other evolutionary constraints^135^ may limit the capacity for adaptive evolution at times. Consistent with constraints on adaptive evolution, there is growing evidence that antibiotic-resistant strains commonly arise via strain replacement after treatment rather than *de novo* resistance evolution in existing strains.^156^

### Evolutionary adaptation within microbiomes

When a new nutrient appears in a microbiome, microbes may be able to adapt phenotypically with transcriptional reprogramming that shifts their metabolism.^153^ However, in other cases, there will be natural selection for genetic changes and evolutionary adaptation to the prevailing conditions. In support of this expectation, the prevalent fiber-degrader *Bacteroides thetaiotaomicron* rapidly evolved in mice on a low-fiber diet to increase its mucus utilization.^157^ Natural selection is also expected to favor shifts in metabolism when strains overlap strongly in their nutrient requirements. If both strains manage to avoid extinction, this process of character displacement can allow two formerly competitive strains to move into separate niches and coexist^151,158^ (Figure 6B). Consistent with the potential for character displacement, recent preliminary work indicates that *Clostridioides difficile* metabolism will evolve from proline to glucose utilization in laboratory experiments in a manner that reduces competition with other species.^159^ Similar patterns were seen in earlier studies that allowed environmental communities of microbes to evolve in the lab.^160,161^ Character displacement, therefore, may be a central process driving the evolution of metabolism within microbiomes.

However, natural selection can also amplify the effects of nutrient competition via the evolution of bacterial warfare.^88^ There is evidence, for example, that *Staphyloccus aureus* variants evolve during infections to overproduce bacteriocins that inhibit competing strains.^91^ When weapon genes are transferred horizontally between strains, nutrient competition is again important for the outcome. If donor and recipient strains are metabolically similar and compete strongly for nutrients, a recipient of weapon genes is unlikely to establish. However, when the two strains have distinct nutrient niches, both can prosper, thereby cementing the transfer of the bacterial weapon to a new genotype.^162^

The evolution of cross-feeding is easy to understand when it arises via a metabolic byproduct of one strain that another uses (Figure 6C). Less easy to explain in microbes is the evolution of two-way cooperation (+/+), where both species help one another. Both evolutionary theory and experiments suggest that these interactions are fragile and rest upon specific requirements.^6,163^ These requirements include that the two species do not compete strongly over limiting nutrients and can reliably find—and interact preferentially with—the cooperative members of the other species, which in a diverse community is a challenging prospect.^163^ Evolutionary theory, therefore, suggests that the amino acid auxotrophies observed in many bacteria^74^ typically do not evolve as specific pairwise cooperative relationships between species. Instead, they may evolve when there is a general availability of free amino acids in an environment, be it from cell lysis in the microbiota, the host, or ingested food. However, more experimental work is required to fully understand the evolution of these interesting auxotrophies.

The observed types of ecological interactions within microbiome communities, therefore, are expected to readily emerge from natural selection on strains to utilize the available nutrients. By maintaining an anaerobic environment and favoring species that ferment complex carbohydrates, an animal host can generate ecological and natural selection for gut communities that are diverse, stable, and metabolically able to break down the nutrients that it cannot.

## Outlook and applications

We have argued that the map between nutrient availability and microbial metabolism is central to the ecology, evolution, and impacts of microbiomes. However, there remain large gaps in our understanding of metabolic ecology. There is a need for new ecological theory that better bridges the metabolic and ecological scales, which are currently often modeled independently, e.g., via flux balance analysis and Lotka-Volterra equations, respectively.^4^ A compromise between such approaches is achieved with consumer-resource models,^164^ which incorporate nutrient competition by explicitly capturing microbial species and consumed nutrients. These methods sometimes remain interpretable via analytical techniques when the number of nutrients is small, such as the Tilman’s graphical approach (Figures 2G–2I).^165^ However, to fully explore the metabolic ecology of microbiomes with consumer-resource models, new techniques are needed that can (1) parametrize these models based upon the metabolic networks of the consumers and (2) extend the analytical approaches to multiple nutrients, for example, by approximating the consumer-resource models with appropriately parametrized Lotka-Volterra models.^63,166^ Such analytical techniques will allow us to understand better how key properties of microbial communities, such as their assembly, invasibility, or stability, result from the metabolic networks of the constituent species (Figure 4).

Empirically, we need to study the nutrient landscape in microbiomes in both time and space, including at the micron scales at which microbes often interact.^43^ Here, untargeted mass spectrometry methods can survey metabolites,^167^ while techniques that combine isotope labeling with metabolomics, proteomics, or Raman microspectroscopy can identify whether metabolites are diet or host derived.^49,168^ SmartPills measure pH, temperature, and pressure along the gastrointestinal tract,^169^ while CapScan pills capture a series of samples as they pass down the intestine.^170^ Another interesting approach is using CRISPR to record changes in the gene expression of microbes during metabolic shifts.^171^

Another priority is the study of microbial metabolism. Following a heyday in the last century,^172,173^ the subject now receives less attention. We believe that it is imperative to reverse this trend and refocus on this most fundamental of phenotypes. We need to better understand the nutrient preferences of individual microbes, both alone and in communities.^13,18,19,39^ These preferences are sometimes predictable based on the energy that a nutrient provides, but not always, and some species use nutrients simultaneously.^174,175^ Understanding the regulatory mechanisms that underlie these preferences is also of great interest. Sometimes this can occur in surprising ways. For example, recent work found that non-canonical start codons can modulate metabolism and lead to different nutrient preferences among strains.^176^

Many microbiomes are spatially structured both at anatomical and microscopic scales,^43^ and mucosal surfaces can be a resource over which microbes compete.^177,178^ Understanding the effects of spatial structuring on microbiome ecology is an important research frontier. Theoretical work has begun to address this with spatially explicit models coupled to either consumer-resource or flux balance analysis to study the effect of space on a microbe’s ecology and evolution.^179,180^ Consistent with the potential importance of space, experimental work suggests that structuring can enable species to coexist that would otherwise not.^178^ However, the degree of structure in environments like the gut microbiome remains unclear. Here, advances are being made in spatial imaging using fluorescence *in situ* hybridization coupled with methods to spatially preserve microbiome samples.^43,144^ The application of such methods will help to reveal which strains and species actually meet and compete over nutrients. We also need a better understanding of the temporal patterns within communities, including the natural turnovers within a niche that often will be driven by strain-strain interactions rather than interactions between species.^181,182^

A focus on metabolic ecology will help to manipulate and engineer microbiomes. The potential for nutrient blocking^13^ raises the possibility of designing communities of protective microbes using metabolic principles.^183^ A related idea is to augment existing microbiomes to increase metabolic diversity and thereby promote colonization resistance.^184,185^ Similar principles may be leveraged in plants,^186^ where nutrient competition with the microbiota can again suppress pathogens.^187^ As well as blocking pathogen entry, there is the potential to remove resident pathogens from microbiomes, which can be crudely achieved via fecal microbial transplants. These treatments are used therapeutically to suppress *C*. difficile,^188^ whose fate in the gut is linked to nutrient competition.^189,190^ In addition, a recent study showed that both fecal microbial transplants and a defined consortium can be used to inhibit *Klebsiella pneumoniae* in the mouse gut via nutrient competition for gluconate.^191^ Another promising approach is to combine the power of nutrient competition with other mechanisms that suppress pathogens, including phage predation,^192^ antimicrobial toxin-producing bacteria,^123^ and vaccination that raises IgA antibodies specific to the surface glycans of target bacteria.^193^ Here, a recent study found that the ability of a vaccine to eliminate a target strain of bacteria rested upon there also being strong nutrient competition between the target and another strain,^194^ underlining the potential power of such combined approaches.

The successful engraftment of beneficial species also rests upon metabolic principles, because, like pathogens, these species can be subject to nutrient blocking. One important strategy to engraft a beneficial strain is to supplement with a nutrient that only it can use. Here, the stable but reversible engraftment of *B. thetaiotaomicron* in the mammalian gut was achieved by engineering it to utilize porphyrin and then supplying porphyran to generate a private niche.^195^ More generally, the provision of nutrients for microbes has the potential to reshape microbiomes. At present, relatively little is known about how microbiome ecology is influenced by an influx of new nutrients, beyond general shifts in composition.^196^ However, a recent study suggests that the diet given to mice is important for microbiome recovery after antibiotics, where the best recovery was seen with a diet that promoted cross-feeding.^197^ More targeted metabolic interventions are also possible. For example, oxygen levels in the gut can be reduced via compounds that promote the respiration of colonocytes in a manner that helps protect the microbiome from pathogens that use respiration to proliferate.^198^

Microbiomes can be vastly complex systems that affect hosts in diverse ways. However, the ability to harvest and utilize nutrients is fundamental for ecological success in microbial communities, which cements the key link between metabolism and ecology and offers general principles that can be applied to all microbiomes. The study of metabolic ecology holds great promise for understanding the composition, evolution, and functioning of these vital communities.
